# Does Age Affect the Response to Zinc Therapy for Diarrhoea in Bangladeshi Infants?

**Published:** 2008-03

**Authors:** Christa L. Fischer Walker, Robert E. Black, Abdullah H. Baqui

**Affiliations:** Department of International Health, Johns Hopkins Bloomberg School of Public Health, 615 North Wolfe Street, Room E5543, Baltimore, MD 21205, USA

**Keywords:** Age factor, Diarrhoea, Child, Cluster randomized trials, Community-based studies, Diarrhoea, Infantile, Infant, Zinc, Zinc supplementation, Zinc therapy, Bangladesh

## Abstract

The benefit of zinc for the treatment of diarrhoea in a cluster-randomized trial of children, aged 3–59 months, living in rural Bangladesh was previously reported. Here, the benefits of zinc stratified by age—3–5 months, 6–11 months, and 12–59 months—are reported. Although the sample sizes in the stratified groups were too small to detect statistical significance in the 3–5-month and 6–11-month age-groups, the trends suggest that there may be a benefit of zinc for the treatment of diarrhoea on the duration of diarrhoea and on subsequent morbidity and mortality. Additional research is needed to better understand the effect of zinc for the treatment of diarrhoea among infants aged less than six months.

## INTRODUCTION

Zinc is an essential micronutrient for human growth and immune function (1). When given as an adjunct therapy for the treatment of diarrhoea, zinc decreases the duration and severity of the episode (2,3). In addition, supplementation of zinc for 10–14 days given during a diarrhoea episode has been shown to decrease diarrhoea and acute lower respiratory infections (ALRIs) in the 2–3 months following the episode (2,4).

The World Health Organization recommends 10–14 days of zinc for the management of diarrhoea among children aged less than five years, in addition to oral rehydration solution (ORS) (5). Data supporting zinc for the treatment of diarrhoea is extensive for children aged six months to five years. However, only two studies have been conducted exclusively among infants aged less than six months and found no effect on the diarrhoea episode or on subsequent morbidity (6-8).

Of trials that have enrolled infants as young as three months, a few have stratified by age, and none has categorized the differences between infants aged less than six months and those aged ≥6 months. We previously reported the results of a cluster-randomized trial of zinc supplementation for the treatment of diarrhoea that included 11,772 child-years of observation (2). Here, we present the effects of zinc on the duration of diarrhoea, incidence, hospitalizations, and mortality in a stratified analysis of infants aged 3–5 months and 6–11 months and compare these results with those observed in children aged 12–59 months.

## MATERIALS AND METHODS

### Study side and study subjects

Thirty areas under the community health worker (CHW) were randomized to introduce zinc supplementation (20 mg of zinc acetate per day for 14 days), in addition to ORS for the treatment of diarrhoea or ORS alone, the standard of care at the time of the study. The details of the study design, randomization procedure, and follow-up have been previously reported (2). Briefly, we enrolled children, aged 3–47 months, living in the rural Matlab field area of the International Centre for Diarrhoeal Disease Research, Bangladesh (ICDDR,B) at the start of the study. Children living in the intervention and control villages continued to be enrolled as they reached three months of age and were included in follow-up until the age of 59 months. All children were visited once every two months to collect data on diarrhoea (one-week recall) and ALRI morbidi-ty (two-week recall). Diarrhoea was defined as ≥3 loose, liquid, or watery stools in 24 hours. ALRI was diagnosed if the infant had reported cough, difficult breathing, and rapid breathing or chest in-drawing. A random sample of 1,252 diarrhoea episodes was followed up to determine the duration of the episode. However, the total sample among infants aged 3–5 months was quite small because of the narrow age band and the overall lower rates of diarrhoea among infants aged less than six months. All hospitalizations were recorded from the Matlab Health Research Centre of ICDDR,B, and each child death from the study villages was identified by an ongoing health and demographic surveillance system. As this was a community-based study and zinc was only available for the treatment of diarrhoea, only children with a diarrhoea episode since the start of the study would have received zinc.

Consent was obtained from all participating villages during community meetings to explain the study. Individual informed consent was also obtained from parents of surveyed children prior to partaking in a survey. The Ethical Review Committee of ICDDR,B approved the study design and consent procedures.

### Statistical methods

Selected characteristics of infants at enrollment were compared between those living in intervention versus comparison clusters. In age-stratified analyses, the duration of diarrhoea was compared between infants living in the intervention and comparison clusters using individual-level Student's *t*-tests and Cox's proportional hazards ratios adjusted for age, sex, birth-order, and size of household homestead land area (9). Incidence rates of diarrhoea and ALRI were calculated by dividing the periods observed with diarrhoea or ALRI by the total periods of observation (x 100 child-years). Rates of hospitalizations were calculated by dividing the total admissions by the number of child-years observed (x 100 child-years). Rates of non-injury deaths were calculated by dividing the number of deaths by the number of child-years observed (x 1,000 child-years). The differences in mean rates and corresponding confidence intervals between children living in the intervention and the comparison clusters were then calculated using cluster-level *t*-test analyses. All analyses were performed with the Stata software (version 9.0) (9).

## RESULTS

Age, sex, and birth-order were similar between children living in the intervention and those living in the comparison clusters at the start of the study among all children (data previously presented) and among age-stratified subgroups (2). In a subsample of active diarrhoea episodes, a trend towards a benefit of zinc on the duration of diarrhoea was observed among infants aged 3–5 months (the mean difference 1.6 days), although the benefit of zinc on the duration of diarrhoea was only statistically significant among older children (p<0.001 and hazard ratio=0.77, 95% confidence interval: 0.68–0.87 if >12 months) (Table 1). There was a non-statistically significant trend towards lower rates of ALRI incidence among infants in each age-stratum living in the zinc intervention compared to the comparison clusters as observed from cross-sectional community surveys (Table 2). The rates of diarrhoea incidence were lower among children living in the intervention clusters compared to the comparison clusters for those aged 6–11 months (the difference in incidence rate [IR]=3.36; p<0.05) and aged >12 months (the difference in IR=2.86; p<0.05). There was a trend towards lower rates of ALRI and diarrhoea hospitalizations among infants aged 3–5 months and 6–11 months living in the intervention clusters vs the comparison clusters and a statistically significant reduction among children aged >12 months. Children living in the zinc clusters in each age stratum had lower rates of non-injury mortality compared to children living in the comparison clusters, although these were not statistically significant when stratified by age (Table 3).

**Table 1:** Duration of diarrhoea episodes* by intervention group among a sample of active diarrhoea cases in Matlab, Bangladesh

Age (months)	Intervention		Comparison			
	No.	Days	No.	Days	p value‡	Hazard ratio (95% CI)
3–5	16	6.4±4.3†	22	8.0±6.3	0.3865	0.76 (0.39–1.47)
6–11	84	5.8±4.6	83	6.8±4.9	0.1757	0.82 (0.61–1.12)
<12	100	5.9±4.5	105	7.1±5.2	0.0794	0.80 (0.61–1.06)
12–59	520	4.5±3.5	527	5.7±4.5	<0.001	0.77 (0.68–0.87)

*≥3 loose or watery stools in 24 hours

†Data are presented as mean±standard deviation

‡Differences in means between intervention and comparison assessed by Student's *t*-test analysis; CI=Confidence interval

**Table 2:** Rates of ALRI and diarrhoea incidence* and of hospitalization in intervention vs comparison clusters in Matlab, Bangladesh

Outcome variable	3–5 months			6–11 months			12–59 months		
	Intervention	Comparison	Differences in mean rates (95% CI)**	Intervention	Comparison	Differences in mean rates (95% CI)**	Intervention	Comparison	Differences in mean rates (95% CI)**
ALRI incidence									
Periods observed	547	578		2,752	2,862		17,400	17,649	
Periods with ALRI	50	55	0.38	246	289	1.16	1,247	1,356	0.56
Unadjusted incidence	9.14	9.52	(−3.17–3.90)	8.94	10.10	(−0.43–2.75)	7.17	7.68	(−0.06–1.09)
Diarrhoea incidence									
Periods observed	547	578		2,752	2,862		17,400	17,649	
Periods with diarrhoea	118	125	0.06	642	764	3.36	2,518	3,058	2.86
Unadjusted incidence	21.57	21.63	(−4.97–5.08)	23.33	26.69	(1.00–5.73)1	14.47	17.33	(2.06–3.66)‡‡
Hospitalization due to ALRI									
Child-years observed	328.5	342.3		690.6	707.9		4,846.9	4,964.8	
Admissions	36	41	1.02	78	83	0.43	182	236	1.00
Unadjusted admission rate	10.96	11.98	(−3.99–6.08)	11.29	11.72	(−3.06–3.93)	3.75	4.75	(0.41–1.59)‡‡
Hospitalization due to diarrhoea									
Child-years observed	328.5	342.3		690.6	707.9		4,846.9	4,964.8	
Admissions	61	69	1.59	189	223	4.16	228	330	1.95
Unadjusted admission rate	18.57	20.16	(−4.61–7.88)	27.34	31.50	(−0.84–9.13)	4.70	6.65	(0.99–2.90)‡‡

*Rates of diarrhoea and ALRI incidence were calculated from bi-weekly cross-sectional surveys

**Based on cluster-level *t*-test analysis. The differences in mean rates expressed per 100 child-years of observation

†ALRI was defined by the following symptoms: cough, difficult breathing and rapid breathing, or chest in-drawing

‡Statistically significant benefit of zinc on the incidence of disease and hospitalization (p<0.05)

ALRI=Acute lower respiratory infection; CI=Confidence interval

**Table 3:** Non-injury deaths rates among intervention and control clusters in Matlab, Bangladesh

Age stratification for noninjury deaths	Intervention	Comparison	Differences in mean rates (95% CI)*
3–5 months			
Child-years observed	328.5	342.3	
Non-injury deaths	3	8	
Non-injury death rate	9.1	24.7	15.6 (−5.3–36.4)
6–11 months			
Child-years observed	690.6	707.9	
Non-injury deaths	6	7	
Non-injury death rate	8.7	9.9	1.2 (−8.5–10.9)
<12 months			
Child-years observed	1,019.1	1,032.2	
Non-injury deaths	9	15	
Non-injury death rate	8.8	14.5	5.7 (−4.0–15.4)
12–59 months			
Child-years observed	4,846.9	4,964.8	
Non-injury deaths	4	12	
Non-injury death rate	0.8	2.4	1.6 (−0.1–3.3)

**Based on cluster-level *t*-test analysis. The differences in mean rates expressed per 1,000 child-years of observation

## DISCUSSION

In this stratified analysis of children enrolled in our large cluster-randomized effectiveness trial, we observed a trend towards a benefit of zinc for diarrhoea on the duration of diarrhoea and subsequent morbidity and mortality in each age-stratum (3–5 months, 6–11 months, and 12–59 months). The sample sizes for this study were originally calculated to detect a 33% difference in hospitalization rates and a one-day difference in the duration of diarrhoea episodes among children aged 3–59 months in the intervention clusters vs the comparison clusters. Although the original study design was not powered to detect statistically significant differences for the main outcome measures when stratified by age, we conducted this secondary analysis because there have been only two studies of zinc for the treatment of diarrhoea that enrolled infants aged less than six months. Since these two recently-published randomized placebo-controlled trials of infants aged less than six months found no difference in the duration or severity of diarrhoea between infants who received zinc or those who received placebo (6,7), we felt it important to determine if the same trend among young infants was observed in this large cluster-randomized trial.

Unlike the two previously-published studies, we observed a trend for shorter duration of diarrhoea among infants in the zinc clusters which was similar to that observed in older children. In our community-based study, infants living in the zinc clusters were treated with 20 mg of zinc for 14 days. Brooks *et al*. did not observe any effect of zinc on the duration of diarrhoea among Bangladeshi hospitalized male infants, aged 1–6 month(s), who received either 5 mg or 20 mg of zinc per day during hospitalization (6). Fischer Walker *et al*. did not observe an effect of zinc on the duration of acute diarrhoea among Ethiopian, Indian and Pakistani infants, aged 1–5 months, who received 10 mg of zinc per day for 14 days upon presentation to an outpatient clinic (7). It is possible that the differences in populations and dose may account for the variation in results. Although zinc has been shown to benefit hospitalized children (10,11), young infants with episodes requiring hospitalizations may have additional complications that mask the benefit of zinc supplementation. The current recommendations of WHO call for 10 mg of zinc per day for 10–14 days for infants aged 1–5 month(s) as was given in the study by Fischer Walker *et al*. (7). Given the lack of effect observed in this study with 10 mg of zinc per day and our positive trend giving 20 mg per day, it is possible that the 20-mg dose recommended for older children may also be beneficial for young infants. A 20-mg dose has been shown to be safe (6). However, additional research may be important to verify these safety results and further evaluate possible efficacy among infants aged less than six months.

Zinc for the treatment of diarrhoea has been shown to decrease subsequent morbidity due to diarrhoea and ALRI in older children but not among infants aged less than six months (4,8). Similar to what has been previously reported, we observed a significant benefit of zinc for diarrhoea on the future incidence of diarrhoea only among children aged ≥6 months. However, this trial is different from the previously-published efficacy trial in this age-group because, in this study, data on morbidity were collected from random surveys which might have included infants who had not yet been treated for a diarrhoea episode (the only opportunity to have received zinc) at the time of the survey.

The benefit of zinc on hospitalization rates was only statistically significant among children aged ≥12 months, although the absolute differences in rates were not the greatest in this age-group, suggesting that the lack of observed effect among these infants may be a result of inadequate sample size. We previously reported an overall 51% reduction in mortality among infants living in zinc clusters (2). Although the statistical significance was lost in the stratified analyses, the trend among infants aged less than six months was consistent with the overall reduction in morbidity.

Although young infants typically experience fewer episodes of diarrhoea than older children, it is of public-health importance to understand if zinc is effective for the management of diarrhoea in infants aged less than six months. To date, randomized efficacy trials have not demonstrated a benefit or risk of zinc for the management of diarrhoea among infants aged less than six months. Although the results of this stratified analysis lacked the power to demonstrate a statistically significant effect, the reductions we observed were clinically similar to those we reported in all children and suggest that young infants may benefit from zinc therapy given at 20 mg per day for the management of diarrhoea. Additional research and analyses of existing data are needed to determine if this trend has been observed in other large trials which included young infants aged less than six months.

## ACKNOWLEDGEMENTS

The study was supported by Johns Hopkins Family Health and Child Survival Cooperative Agreement and ICDDR,B Cooperative Agreement with funding from the United States Agency for International Development.
